# pMD-Membrane: A Method for Ligand Binding Site Identification in Membrane-Bound Proteins

**DOI:** 10.1371/journal.pcbi.1004469

**Published:** 2015-10-27

**Authors:** Priyanka Prakash, Abdallah Sayyed-Ahmad, Alemayehu A. Gorfe

**Affiliations:** University of Texas Health Science Center at Houston, Department of Integrative Biology and Pharmacology, Houston, Texas, United States of America; George Mason University, UNITED STATES

## Abstract

Probe-based or mixed solvent molecular dynamics simulation is a useful approach for the identification and characterization of druggable sites in drug targets. However, thus far the method has been applied only to soluble proteins. A major reason for this is the potential effect of the probe molecules on membrane structure. We have developed a technique to overcome this limitation that entails modification of force field parameters to reduce a few pairwise non-bonded interactions between selected atoms of the probe molecules and bilayer lipids. We used the resulting technique, termed pMD-membrane, to identify allosteric ligand binding sites on the G12D and G13D oncogenic mutants of the K-Ras protein bound to a negatively charged lipid bilayer. In addition, we show that differences in probe occupancy can be used to quantify changes in the accessibility of druggable sites due to conformational changes induced by membrane binding or mutation.

## Author Summary

We introduce a simulation-based method to identify allosteric ligand binding sites in membrane-associated proteins for which existing methods are inadequate. We applied the method on two mutant forms of an oncogenic protein called K-Ras. We show that the way in which the protein interacts with membrane is an important determinant for the accessibility of selected ligand binding sites. We also describe techniques to quantify changes in the ligand binding potential of cavities on the surface of proteins induced by mutation or membrane binding.

## Introduction

Identification of a suitable ligand-binding site on a drug target is a crucial first step in structure-based computer aided drug discovery [1]. This is not a trivial task if the desired target site is an allosteric one that is not readily observable in average experimental structures [2]. Recently, a number of techniques have been developed that allow for the identification of (allosteric) ligand binding sites in target proteins [3–6]. Because ligand binding site identification usually requires sampling of the target’s configurational space, considerable effort has also been made toward integrating molecular dynamics (MD) simulation into the site identification process (e.g. [6]). In particular, MD-based computational solvent mapping [7–12] is attracting wide attention as a convenient means of binding site identification in dynamic targets. Interest in this approach will likely increase with the expanding scope of MD simulations and because it recapitulates multi-solvent crystallographic [3] and fragment-based NMR screening experiments [5].

A typical MD-based computational solvent mapping entails carrying out MD simulations in the presence of small organic molecules in the solvent (e.g. [7,8]). The goal is to use the small organic molecules as probes to search for binding sites on an ensemble of MD-sampled target structures. The probability of contact (or interaction) between probe and protein atoms is then used to evaluate the druggability of sites. The method has been described in a number of recent reports under various names: probe-based MD [8], mixed-solvent MD [12], solvent competition [7], co-solvent MD [10] and ligand competitive saturation [9,13]. We use the term probe-based MD (pMD) throughout this report. Surprisingly, thus far pMD has been applied only to soluble proteins despite the fact that some of the most important drug targets require membrane binding for their biological activity [14–19]. A major goal of the current work is to extend the applicability of pMD to membrane-bound drug targets. This requires mitigating possible effects of the probe molecules on membrane structure and dynamics. For example, we previously found that small organic molecules such as ibuprofen, indomethacin and cholic acid partition into the hydrophobic core of DPC micelles [20–22]. Others found that similar small organic molecules partition into bilayers [23,24]. Here we describe pMD-membrane, a method that avoids membrane partitioning of probe molecules and enables allosteric ligand binding site identification in proteins bound to a bilayer surface.

Another challenge in current efforts of computational binding site identification is the difficulty in discriminating between closely related homologs or mutations that are associated with different disease phenotypes. Whether pMD can capture small changes in the properties of binding sites due to conformational changes induced by membrane-/substrate-binding or mutation has not been examined. We introduce analysis techniques to evaluate differential probe occupancy that inform on the changes in potential druggability of a site.

We tested pMD-membrane and the new analysis tools on G12D and G13D mutants of K-Ras. We chose these K-Ras mutants as model systems for a number of reasons. First, K-Ras is a prototypical example of membrane-associated small GTPases for which there exist abundant experimental structure data [25]. Secondly, we recently found that the interaction of K-Ras with membrane involves at least two distinct conformations (Prakash and Gorfe, unpublished results). Third, K-Ras is a key regulator of numerous signaling pathways involved in cell division and proliferation [25–27], and therefore it is physiologically and therapeutically highly relevant. In fact, 15–25% of all cancer cases are associated with mutations in the homologous K-, N- and H-Ras proteins [28]; K-Ras mutations represent 85% of these [29].

Previous efforts to inhibiting aberrant Ras function have failed [30,31], but a number of allosteric Ras ligands have been discovered recently [32–38]. While these ligands are promising starting points, none have the necessary potency and selectivity to become a lead compound. Therefore, the search for Ras inhibitors continues. Desirable properties of a potential Ras inhibitor may include the following: (i) Ability to directly bind to membrane-associated cellular Ras. Inhibitor activity in solution is not sufficient because membrane binding is essential for the biological function of Ras, and there is evidence that signaling specificity among isoforms may involve distinct membrane localization and therefore differential accessibility to effectors and modulators [39–42]. It is therefore important that changes in conformation and dynamics upon membrane binding are explicitly considered in binding site identification / drug discovery efforts against Ras proteins. (ii) Specificity toward a given Ras isoform. This is because, as we alluded to above, different Ras isoforms are associated with distinct cancer types [29] despite the fact that they share a catalytic domain that is nearly identical in sequence and average structure [17,43]. For instance, K-Ras mutations are prevalent in lung, colorectal and pancreatic carcinomas [44–47], N-Ras mutations in melanomas and hematologic malignancies [48–50], and H-Ras mutations in bladder and thyroid cancers [51,52]. (iii) Specificity toward a mutation. This is because different Ras mutants, such as G12D and G13D, are associated with different cancer types [25,29,53].

The ability to identify unique ligand binding sites on each Ras isoform or mutant is the first step toward addressing the issues listed above. We demonstrate that pMD-membrane helps achieve this goal, and illustrate its robustness and sensitivity using two of the most common oncogenic mutants of K-Ras: G12D and G13D. We also show that differential membrane binding leads to altered propensity of probes for binding sites.

## Methods

The theoretical basis of pMD can be found in previous reports [7–10], as well as its application to search for allosteric ligand binding sites on K-Ras in solution [11]. Here we focus on technical progresses that allow the use of pMD simulation to predict ligand-binding sites in membrane-bound lipid-modified proteins. We also describe post-simulation analysis techniques to correlate druggability of sites with changes in conformational dynamics induced by mutation or membrane reorientation.

### Generation of initial configurations of membrane-bound mutant K-Ras

**Fig 1:**
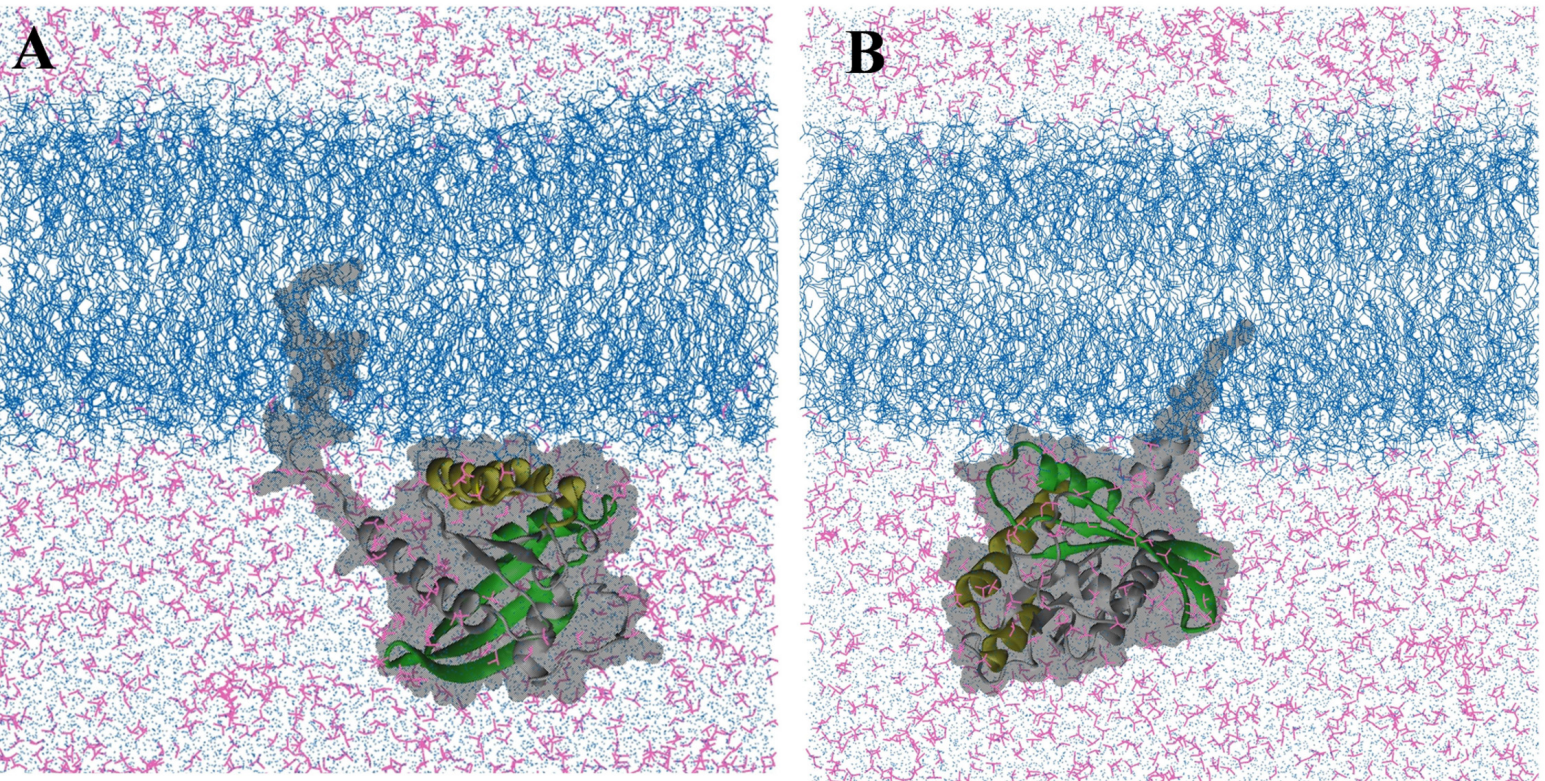
Two dominant orientations of G12D K-Ras in a POPC/POPS bilayer. ***(A)*** Helices 3 and 4 (yellow) directly interact with the bilayer surface (membrane binding mode 1). ***(B)*** Parts of the beta sheet and helix 2 (green) contact the bilayer (mode 2). These conformations have been derived from a microsecond-level MD study of GTP-bound G12D K-Ras in a POPC/POPS bilayer (Prakash and Gorfe, manuscript in preparation). Lipids are depicted as blue lines, the protein as grey ribbon (except for the regions mentioned above) with a surface representation in the background, water molecules are shown as light blue dots, isopropyl alcohol in magenta lines. Ions and hydrogen atoms are omitted for clarity.

Previous simulation [54] and experimental studies [55,56] have shown that the full-length H-Ras protein interacts with membrane in a non-random fashion; it adopts two distinct orientations with respect to the membrane plane depending on the bound nucleotide [57]. In a separate work (Prakash and Gorfe, to be published), we examined the bilayer interaction of GTP-bound G12D K-Ras based on a total of ~7.5 μs all-atom MD simulations. The analysis yielded two predominant modes of membrane binding that differ in the membrane orientation of the catalytic domain (Fig 1). Because these orientation differences can potentially be exploited for the development of isoform- and mutation-specific small molecule inhibitors, we used the two distinct conformations as the starting structure for the current pMD-membrane analysis of G12D and G13D K-Ras, as described below.

### Molecular dynamics simulation of K-Ras mutants in mixed bilayer and mixed solvent

We performed two sets of simulations: reference set and target set. The reference set involved two 60 ns pMD-membrane simulations of G12D K-Ras starting from the two conformations shown in Fig 1. These simulations were performed without any modification to the force field parameters. The target set involved three 100 ns-long pMD-membrane simulations with the Lennard-Jones non-bonded interaction between selected atoms of the probe and lipid molecules modified as described in the following section. Two of the target simulations were on G12D K-Ras while the third was on G13D K-Ras. One of the G12D simulations was started from a conformation in which helices 3 and 4 directly interact with the bilayer (Fig 1A), and the second from a conformation where part of the beta-sheet and helix 2 lie on the surface of the bilayer (Fig 1B). The G13D target simulation was started from the orientation shown in Fig 1A after mutating Gly to Asp at position 13 and reverting the Asp on position 12 back to Gly.

Since cellular K-Ras interacts with negatively charged inner leaflet of the plasma membrane via its polybasic and farnesylated C-terminus, we built a heterogeneous lipid bilayer made up of 320 POPC (1-palmitoyl-2-oleoyl-sn-glycero-3-phosphocholine) and 96 POPS (1-palmitoyl-2-oleoyl-sn-glycero-3-phoserine) lipids. The resulting symmetric bilayer was equilibrated through a 250 ns production simulation. We then embedded full-length G12D or G13D K-Ras4B in one leaflet of the pre-equilibrated bilayer. Membrane insertion was guided by previous reports [58] to determine the insertion depth of the farnesyl tail into the bilayer core. The resulting system was placed in a 114 x 112 x 110 Å^3^ box containing 26299–27573 TIP3P water molecules and 1337–1423 isopropanol probe molecules. In each system, we maintained a 20:1 water to probe ratio and neutral charge by adding 96 sodium ions. Additional sodium and chloride ions were added to mimic physiological ionic strength. The total number of atoms varied between 153316 and 158178 depending on the initial conformation of the protein.

Following system construction and 3000 steps of conjugate gradient energy minimization, we used simulated annealing to homogenize the probe and water molecules around the protein and the bilayer. The annealing process involved the application of a harmonic restraint with a force constant of 4 kcal/mol/Å^2^ on the protein and lipid heavy atoms to prevent protein unfolding and bilayer instability, and incrementing the temperature every 5000 steps by 50 K until a temperature of 650 K was reached, followed by gradual cooling by 10 K every 5000 steps until a final temperature of 310 K was achieved. The resulting system was equilibrated for 1 ns while gradually decreasing the restraint force constant to zero. This was followed by a production run of 60 ns for the reference simulations and 100 ns for the target simulations. A non-bonded cutoff of 12 Å was used during both the equilibration and production phases of each simulation. Long-range electrostatic interactions were calculated by the Particle Mesh Ewald (PME) method [59] with SHAKE [60] restraints applied on bonds involving hydrogen atoms. Simulations were performed with a 2 fs timestep in the NPT ensemble (constant number of particle N, temperature T = 310 K, and pressure P = 1 bar). Nose-Hoover Langevin piston for pressure control was used to maintain constant pressure and Langevin thermostat to maintain constant temperature. Short-range non-bonded forces were computed every timestep and long-range electrostatic forces every other step. All simulations were performed with the NAMD2.9 program [61] using the CHARMM27 force field for proteins [62] and CHARMM36 for lipids [63]; isopropanol was parameterized as described in ref [11].

### Modification of selected non-bonded interaction terms

**Fig 2:**
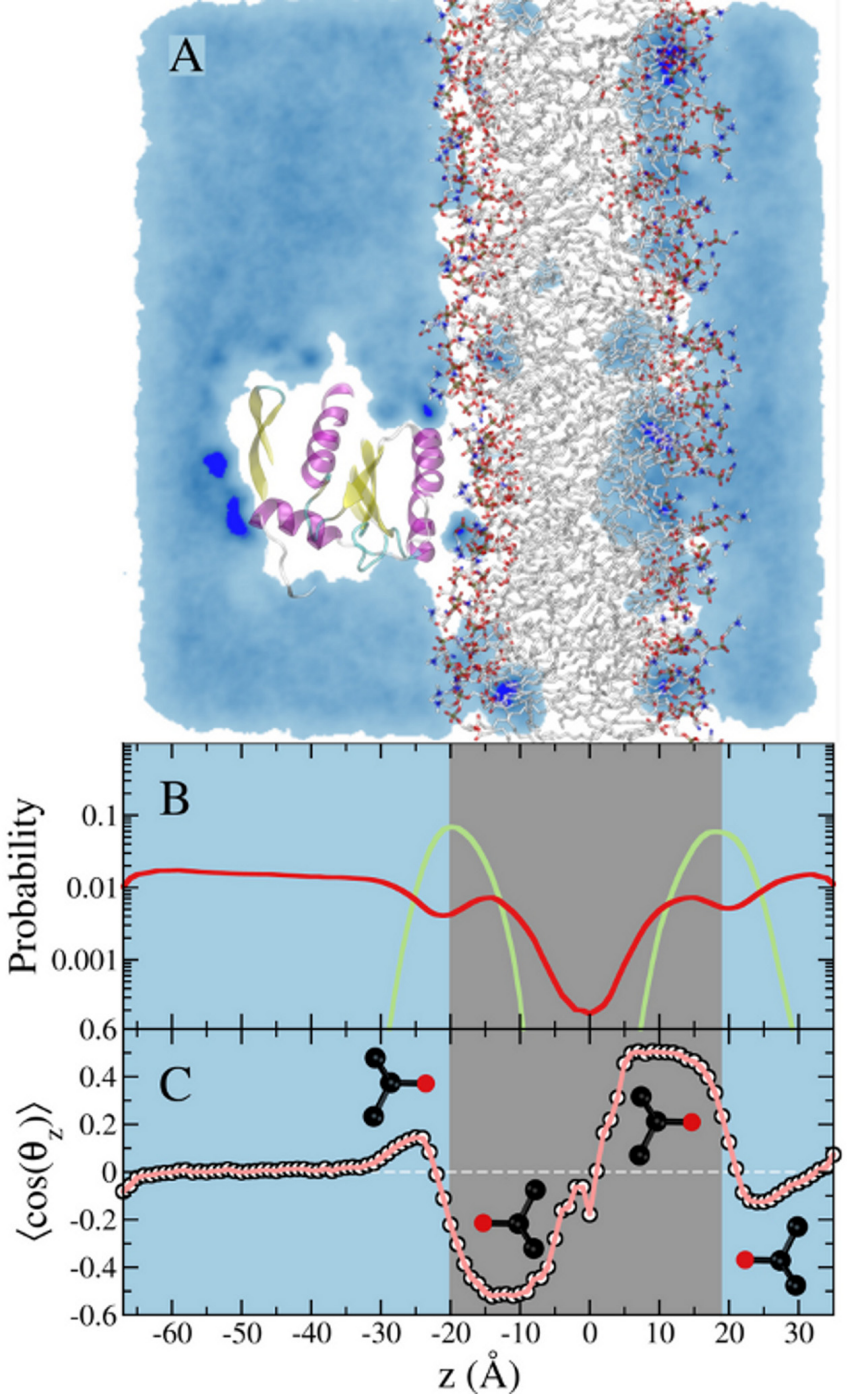
Simulation of membrane-bound G12D K-Ras in the presence of 5% isopropyl alcohol in the solvent. ***(A)*** A snapshot from the simulation showing a cross section of probe density colored in light blue. Probe molecules penetrate the POPC/POPS lipid bilayer (shown in atom colored stick representation) and mainly localize at the interface between the hydrophilic head group region and the hydrophobic tail region of the lipid bilayer. ***(B)*** Density profile of the lipid phosphate group, and the probe center of mass in green and red, respectively. The cyan panel indicates the mixed solvent region, while the gray panel indicates the lipid bilayer region. Notice the bright blue spots on the protein and in the bilayer, highlighting sites where probes are trapped for an extended period(s). ***(C)*** Ensemble-averaged order parameter that characterizes the orientation of the isopropanol probe molecule with respect to the bilayer normal.

pMD-membrane simulation with the unmodified CHARMM force field led to partitioning of a fraction of the probe molecules into the bilayer (see Fig 2 and Results and Discussion). To prevent this diffusion of probe molecules into the bilayer interior, we modified the vdW interaction between the central carbon atom of the probe and the CTL2 atom type of the POPC and POPS lipids. After several tests (see for example S1 Fig), we arrived at the following protocol: the well depth of the Lennard-Jones potential was reduced to a very small value of 0.01 kcal/mol (see ref. 9), and the minimum inter-particle distance among the selected atoms was increased to 7 Å (S1 Fig). The former ensures that attraction between the apolar atoms of the lipids and probe molecules is almost completely eliminated, while the 7 Å distance yields a reasonable balance between allowing the probe to access the polar bilayer surface where the protein sits and preventing it from penetrating into the hydrophobic core (S1 Fig). This modification was made utilizing the NBFIX correction term in the CHARMM force field. This approach is similar in principle to the modifications MacKerell and colleagues made via dummy atoms to prevent aggregation of probe molecules [9].

### Probe occupancy calculations

We calculated the extent of protein-probe interactions using both distance-based and grid-based measures of probe occupancy.

#### (i) Distance-based probe occupancy

The distance-based probe occupancy measure quantifies the probability that a probe molecule is within a certain distance of a given protein heavy atom. Our implementation of this procedure entailed computing the probe occupancy $R_{i}^{x}$ at protein atom *i* in simulation *x* as

$$R_{i}^{x}=\frac{250}{N_{frames}N_{probes}}\sum_{k=1}^{N_{frames}}\sum_{j=1}^{N_{probes}}\frac{1}{1+\alpha d_{ijk}^{n}}$$ (1)

where *N*_*frames*_ is the number of frames in the trajectory, *N*_*probes*_ is the number of probe molecules in the system, *d*_*ijk*_ is the minimum distance between protein atom *i* and any heavy atom of probe molecule *j* at frame *k*. *n* (an integer) and *α* (a small positive real number) are adjustable parameters carefully chosen to include all probes within a desired cutoff value and then smoothly switch off the selection within a small distance range. In this study we used *n* = 20 and *α* = 1×10^−17^ so that all probes within ~6 Å are fully included and those between about 6 and 8 Å are included in a weighted fashion as shown in S2 Fig. We used an arbitrary scaling factor of 250 to facilitate visualization.

To compare changes in the probe-binding potential of a site in two different simulations, we calculated difference probe occupancy (Δ*R*_*i*_) defined as the probe occupancy of atom *i* in trajectory *x* ($R_{i}^{x}$) and the probe occupancy of the same atom in trajectory *y ($R_{i}^{y}$*):

$$\Delta R_{i}=R_{i}^{x}-R_{i}^{y}.$$ (2)

#### (ii) Grid-based probe occupancy

The Grid-based probe occupancy calculation technique has been extensively discussed in previous reports by others [7,8], and has been used in our previous work on K-Ras in solution [11]. In the current work, we calculated grid occupancy in two slightly different ways. In the first approach, only probe molecules that are within a cutoff distance from the protein were included for grid occupancy calculation. We used a cutoff value of 4 Å between any heavy atom of the probe molecule and any heavy atom of the protein. After aligning the trajectory frames based on backbone atoms excluding the flexible switch regions and the termini, we calculated time-averaged number densities per 1 Å^3^ grid using the Volmap and Volutil plugins of VMD [64]. The grid number densities were converted to grid free energies as described previously [11].

In the second approach, we first removed translational and rotational motions by aligning the trajectory to the initial frame of each production run using backbone atoms of the catalytic domain (residues 1–166). Then, a spatial concentration profile of the probe molecules was evaluated on a hexahedral mesh using the following formula

$$c_{ijk}=\frac{1}{dxdydz\left(N_{frames}N_{heavy}\right)C^{0}}\sum_{l=1}^{N_{frames}}\sum_{m=1}^{N_{probes}}\sum_{n=1}^{N_{heavy}}\delta_{\lfloor \frac{x_{lmn}-x_{0}}{dx}\rfloor ,i}\delta_{\lfloor \frac{y_{lmn}-y_{0}}{dy}\rfloor ,j}\delta_{\lfloor \frac{z_{lmn}-z_{0}}{dz}\rfloor ,k}$$ (3)

where *C* ^0^ is the standard state concentration (1*M* = (1 *mole*/*liter* = 1/1660.3 *molecule*/Å^3^), *δ* is the Kronecker delta function, ⌊⌋ is the integer floor function, *c*_*ijk*_ is probe concentration in M at node (*i*,*j*,*k*), *N*_*heavy*_ = 4 is number of heavy atoms in the probe molecule, dx, dy and dz are the hexahedral grid spacing in the x, y and z direction, respectively. We used a uniform grid spacing of 0.37 Å in the spatial concentration profile calculations.

The probe concentration value at each node was smoothed out by assigning an average value based on its neighboring node values,

$$c_{ijk}=\frac{1}{7}\left(c_{ijk}+c_{i-1jk}+c_{i+1jk}+c_{ij-1k}+c_{ij+1k}+c_{ijk-1}+c_{ijk+1}\right)$$ (4)

This averaging procedure eliminates the noise in the constructed spatial concentration profile and assists in making a more vivid iso-surface visualization.

### Analysis of probe orientation

To assess the relative polarity of putative ligand binding sites, we calculated the ensemble-averaged orientation of probe molecules with respect to the surface of the protein. Specifically, we calculated <cos(θ_r_)>, where θ_r_ is defined as the angle between the vector radiating from the central carbon atom to the O atom of an isopropanol probe molecule and the vector connecting the center of the catalytic domain of the protein (residues 1–166) and a given atom on the protein surface. Only protein atoms whose probe occupancy was above an empirically determined threshold were considered for this analysis (in this work we used R_i_ ≥ 0.24).

### Convergence and estimation of sampling error

Convergence of protein-probe interactions was evaluated by monitoring the time evolution of the atom-averaged probe occupancy *R*_*ave*_

$$R_{ave}=\frac{\sum_{1}^{M}R_{i}}{M}.$$ (5)

where the summation is over all protein atoms *M* with *R > 0*.05. The 0.05 cutoff ensures that approximately all of the atoms with non-zero R-value are included in the statistics. (Note that the profile of *R*_*ave*_ would be unaffected even if atoms with zero R-values were also included.) S3 Fig (left) shows that *R*_*ave*_ has equilibrated within the first ~20 ns in all three of our target simulations. Moreover, comparison of the mean (<*R*_*ave*_>) in 20 ns blocks yielded very small differences among all blocks except the first one. For instance we obtained <*R*_*ave*_>±*S*.*D* of 0.20±0.05, 0.21±0.02, 0.22±0.04, 0.24±0.02 and 0.24±0.03 for the 1–20, 20–40, 40–60, 60–80 and 80–100 ns blocks of the G12D simulation in membrane binding mode 1; similar results were obtained for G12D mode 2 and G13D simulations. This suggests that the simulations were well equilibrated in terms of probe binding, and that any portion of the last 80 ns data can be used to compute average occupancies. For a better statistics, however, we used all frames in the last 80 ns of the trajectories unless stated otherwise.

Statistical uncertainty in R_i_ was estimated based on an analysis of block standard error (BSE) following the procedure described by Grossfield and Zuckerman [65]. In this analysis, BSE was calculated at different sizes of time blocks (*b*_*n*_). The convergence of BSE versus *b*_*n*_ was used to evaluate the quality of our sampling and to determine the maximum value of the BSE, which serves as a measure of our sampling error. In S3 Fig (right), we show several BSE vs. *b*_*n*_ plots for a few atoms chosen for illustration of the diverse convergence rates and error values. For a more rigorous analysis of errors in the relative probe accessibility of each atom in trajectories *x* and *y*, i.e., the uncertainty in *ΔR*_*i*_, we first calculated BSE in $R_{i}^{x}$ and $R_{i}^{y}$ for multiple block sizes *b*_*n*_. We then used nonlinear fitting of the dependence of BSE on *b*_*n*_ to the following functional form: *a*_0_(1-*a*_1_ *exp*(-*a*_2_ *b*_*n*_)), where *a*_0_, *a*_1 and_ *a*_2_ are three fitting parameters with *a*_0_ being an estimate of the asymptotic value of BSE. We then computed the uncertainty in *ΔR*_*i*_ by combining the BSEs in $R_{i}^{x}$ and $R_{i}^{y}$ (Eq 6).

$$\delta \left(\Delta R_{i}\right)=\text{BSE}\left(R_{i}^{x}\right)+\text{BSE}\left(R_{i}^{y}\right)$$ (6)

## Results/Discussion

As noted previously, probe-based MD has been successfully applied to a number of soluble drug targets [8–10,12], including the soluble catalytic domain of K-Ras [11]. Yet in cells K-Ras and a large number of other targets are membrane-bound. Therefore, we first describe the challenge of using probes in membrane simulations and how to overcome the challenge. We will then discuss the application of pMD-membrane on G12D and G13D K-Ras proteins in bilayer.

### Preventing partitioning of probe molecules into bilayer

To our knowledge pMD has not been previously applied to membrane proteins. Because molecular probes are completely or mostly nonpolar and small, we reasoned that one of the challenges might be the possibility that they partition into and modulate the structure of the bilayer. To test this hypothesis, we ran two 60 ns pMD-membrane simulations without the Lennard-Jones non-bonded interaction modifications described above. We found that a significant fraction of our test probe, isopropanol, quickly partitioned to the POPC/POPS bilayer (Fig 2A). Moreover, the interaction of the probe molecules with the membrane lipids is non-random (Fig 2A). This is quantified by the number density distribution of the probes along the bilayer normal (Fig 2B), where the peaks within the bilayer indicate preferential accumulation near the glycerol region. To complement this observation, we calculated the average orientation of the probe molecules along the bilayer normal based on the cosine of the angle between a vector along the bond connecting the central carbon and the hydroxyl oxygen of the probe molecule and the membrane normal. As shown in Fig 2C, the OH moiety of the probe appears to be donating a hydrogen bond to the carbonyl oxygen of lipid glycerol. Thus, in contrast to the random mixing of the probe with water (see the blue shade in Fig 2A), isopropanol-lipid interaction is specific, as illustrated in Fig 2C. As a result, the average area per lipid increased by more than 10% (60.8 ± 0.7 Å^2^ vs. 68.6 ± 3.8 Å^2^ in the absence and presence of probe, respectively). Correspondingly, the bilayer thickness decreased from 40.5 ± 0.3 Å to 38.0 ± 1.4 Å.

**Fig 3:**
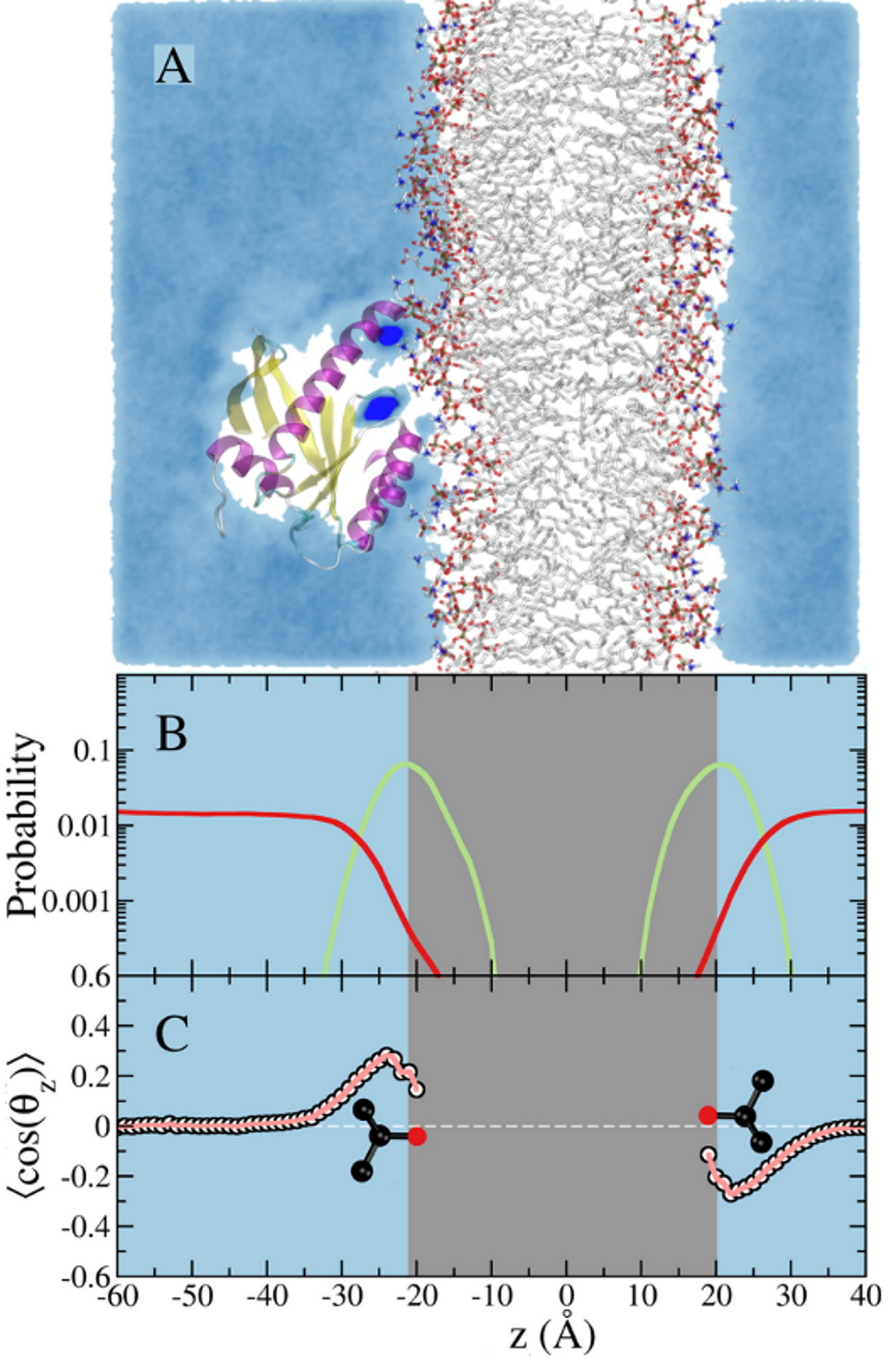
Simulation of membrane-bound G12D K-Ras in the presence of 5% isopropyl alcohol in the solvent with a repulsive potential between the central atom of the probe and the hydrophobic atoms of the lipids. ***(A)*** A snapshot from the simulation showing a cross section of probe density colored in cyan. Probe molecules do not penetrate the POPC/POPS lipid bilayer in this case. Dark blue spots on the protein highlight sites where probes are trapped for an extended period. ***(B)*** Density profile of the lipid phosphate group (green) and probe center of mass (red). ***(C)*** Ensemble-averaged order parameter that characterizes orientation of the probe molecule with respect to the bilayer normal. The color code is the same as in Fig 2.

While the impact of the isopropanol-lipid interaction on the bilayer structure might appear relatively small, it can have substantial impact on the dynamics of the bound protein. Moreover, we anticipate larger effect for bigger and more lipophilic probes such as benzene or cyclohexane. Therefore, it is desirable to avoid probe partitioning into the bilayer in order to ensure that pMD-membrane will have broad application. To achieve this, we modified the Lennard-Jones potential between selected atoms of the probe and lipids (see Methods). As shown in Fig 3, this modification led to a smooth decline in the number of probe molecules that approach the polar head group, and exclusion of probe molecules from the hydrophobic core. This is reflected in the average area per lipid and bilayer thickness, both of which remained unaffected (59.7 ± 0.4 Å^2^ vs. 60.8 ± 0.7 Å^2^ and 41.2 ± 0.2 Å and 40.5 ± 0.3 Å in the presence and absence of probes, respectively). The very small polarization of the few probe molecules near the bilayer surface (Fig 3C) can be eliminated if needed by using a larger non-bonded inter-particle distance for the modified atom pairs. Our choice of parameters was meant to ensure that probe molecules can approach the protein from the side of the membrane surface as well as from bulk.

In summary, comparison of Figs 2 and 3 makes it clear that a simple modification of some of the vdW terms on selected atoms of the probe and bilayer extends the applicability of pMD to membrane proteins, a major focus of many drug discovery campaigns (e.g. [19]). In the subsequent sections we demonstrate the application of the method on membrane–bound K-Ras, a highly sought after anti-cancer drug target [30,66].

### Comparison of druggable sites on K-Ras predicted by pMD in membrane and in solution

Protein motion can be affected by the composition of the surrounding solvent [67,68]. As can be surmised from Fig 1, the dynamics of the catalytic domain of K-Ras G12D is different before and after it formed direct contact with the bilayer (i.e., when fully in water and after part of the surface is restrained by interaction with lipids). Therefore, we checked if (i) pMD-membrane qualitatively reproduces binding sites on Ras that have been previously characterized by other solvent mapping techniques [69,70], and (ii) these sites/sub-sites are modulated by conformational change induced by membrane binding.

In the previous pMD analysis of G12D in solution [11], we identified five druggable sites and three sub-sites (see Fig 3 in ref. [11]). A detailed comparison with known ligand binding pockets indicated that three of the predicted druggable sites overlap well with pockets p1, p2 and p3 while two of the predicted sub-sites were found to be parts of p1 and p4, respectively (see Fig 4 of ref. [11]). Allosteric pockets p1 to p4 have been previously described in detail: p1 is the binding site of ligands reported by Maurer et al [32], Sun et al [33] and Shima et al [34]; p3 is near the C-terminus of the catalytic domain where Cu^2+^-cylen binds [35,36]; p4 is the proposed target site of Andrographolide derivatives [38] and Zn(II)-bis(2 picolyl)amines [36]; there is no known non-covalent binder that targets p2 but covalent ligands that target this region have been reported [37]. Direct comparison of the calculated probe occupancies in membrane-bound K-Ras with experimental results is not possible because one cannot turn on in experiment non-physical repulsive interactions to prevent bilayer partitioning of isopropanol. Nonetheless, the following analysis provides a strong evidence that pMD-membrane is able to identify true drug binding sites.

**Fig 4:**
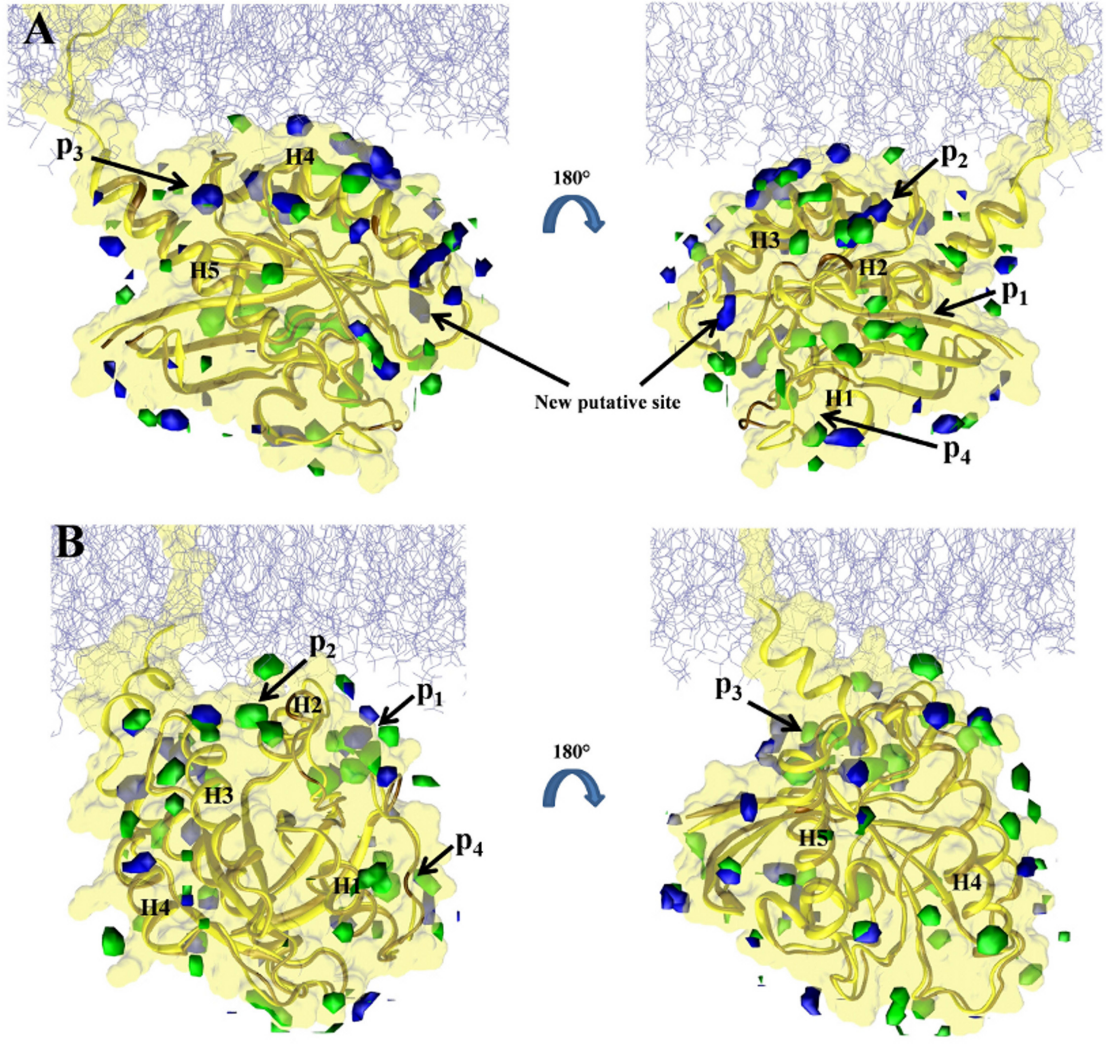
Predicted binding sites in the soluble and membrane-bound G12D K-Ras. Isosurfaces of probe densities with grid free energy values ≤ -1 kcal/mol are shown in solution (green) and membrane (blue) environment. ***(A)*** Membrane binding mode 1. ***(B)*** Membrane binding mode 2. Previously characterized pockets p1 (near β1-β3/h2), p2 (between h2/h3), p3 (near h5 and loop7) and p4 (behind s1) are labeled. The last 10 ns of the current pMD-membrane runs and last 10 ns of the longest run of G12D K-Ras in solution from ref [11] were used to calculate the probe densities and grid free energies. The protein from solution was first aligned to each of mode 1 and mode 2 membrane-bound forms prior to grid-based calculations. The protein is in yellow and a portion of the inner leaflet of the bilayer is shown as iceblue lines. See ref [11] for details.

In order to compare the current pMD-membrane with the previous pMD-solution, we performed grid free energy analysis on the last 10 ns data from the two current simulations of G12D K-Ras and the previous pMD-solution run of G12D K-Ras (we chose the longest, 100 ns run). The results are displayed in Fig 4, where grid densities that yielded grid free energies of -1 kcal/mol or lower are shown in blue and green for the simulations in membrane and solution, respectively. Pockets p1 to p4 are labeled where possible. One can see that there is a remarkable overall agreement between the membrane and solution simulations (see overlaps between the blue and green iso-surfaces). We take this as validation of pMD-membrane, because pockets p1–p4 are all confirmed ligand binding sites for which there exist crystallographic or solution NMR structures of K-Ras in complex with ligands [32–36]. However, there are also clear differences. The most significant differences include the following. (i) In membrane binding mode 1 (Fig 4A), pocket p1 is completely invisible. Instead, a new putative site appears very close to the P-loop but distinct from the nucleotide-binding site. (ii) In membrane binding mode 2 (Fig 4B), p2 is absent but no new site is discovered. Taken together, these results demonstrate that pMD-membrane captures known druggable sites, and that protein-membrane interaction modulates binding site accessibility. Whether the observed differences in some of the sites will translate into differential ligand binding in the soluble and membrane-bound K-Ras is yet to be determined. Nonetheless, this observation highlights the importance of incorporating the effect of membrane in Ras drug discovery efforts.

### Visualizing probe densities–-challenges and proposed solutions

For the analysis in the previous section, we included only probe molecules that lie within 4 Å of protein heavy atoms. In principle, grid occupancy can be calculated over the entire system (see Methods, Eqs 3 and 4), so that all high-density grid points around the protein can be considered. Then, the probe density can be visualized at the desired concentration cutoff. An example of this is shown in S4 Fig. There is a clear overlap between the high-density iso-surfaces and known pockets p1 to p4, as well as the sites highlighted in Fig 4A. However, there are also a large number of other high-density regions that, though unlikely to bind drug-like molecules, clutter the picture.

**Fig 5:**
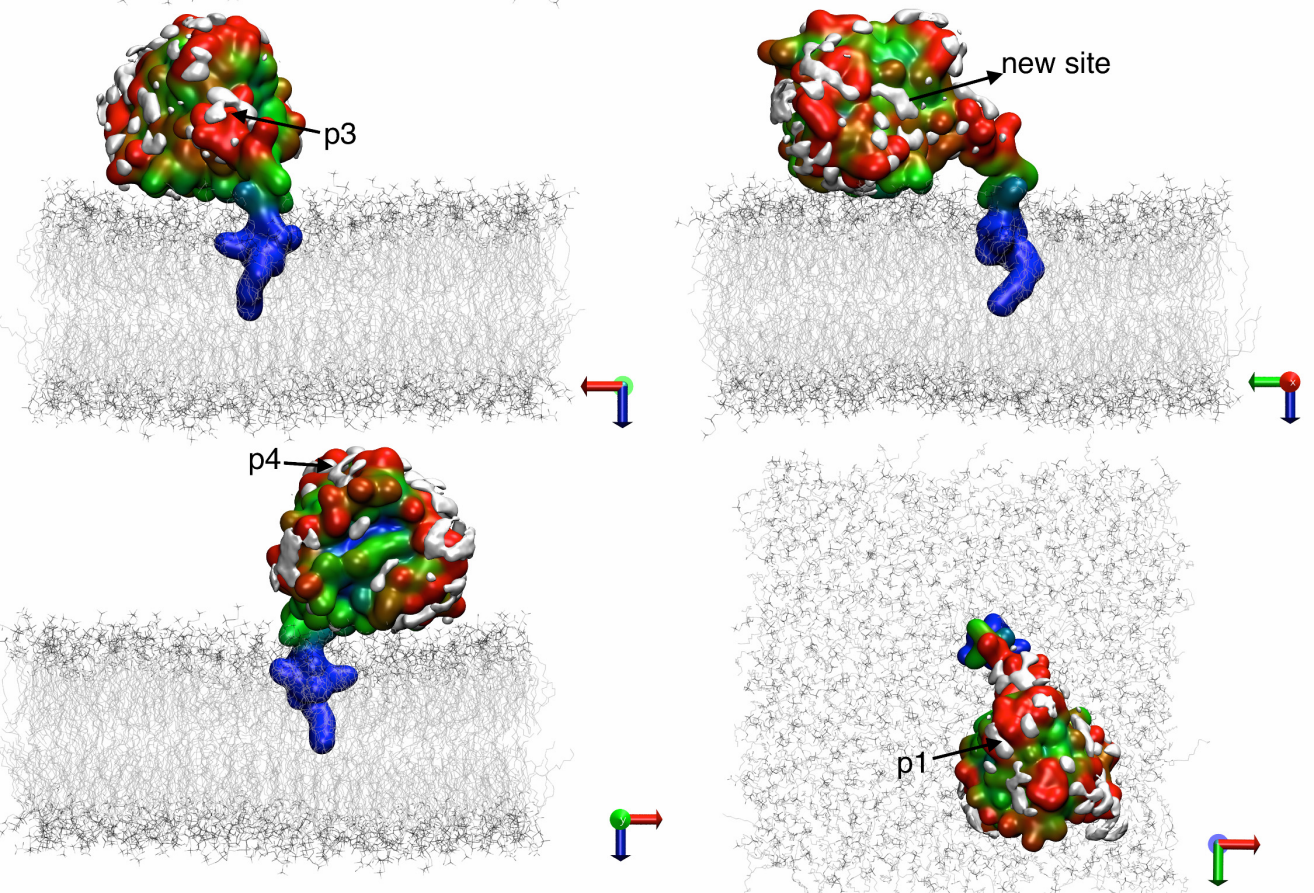
Visualization of probe occupancies from distance-based and grid-based analysis. Shown here are overlays of probe occupancies on K-Ras G12D in membrane-binding mode 1 derived from distance-based and grid-based calculations (see Methods). The bilayer is in grey lines and the protein in surface and is colored in a blue-to-red scale representing low-to-high probe density from the distance-based occupancy measure. The white isosurfaces represent probe density from grid-based calculation.

In Fig 5, we show overlays of probe-occupancies derived from distance-based and grid-based analyses (see Methods). As expected, the two techniques yielded very similar results (notice the overlap between the red and white contours representing high probe densities from distance-based and grid-based analysis, respectively). White isosurfaces circumscribed by the red contours are likely pocket-like, suggesting that a combined use of distance-based and grid-based occupancy analyses would help localize relevant sites somewhat. However, it is still difficult to unambiguously isolate potentially druggable pocket–like sites. This can be regarded as a limitation of probe-based analyses in cases where there is no prior knowledge of druggable sites. This suggests that it is prudent to complement pMD with analysis of geometric/chemical features such as curvature, volume and polarity. There are a number of useful tools to perform structure-based pocket analysis, such as SiteMap [71], MDpocket [72] and AutoGrow [73]. The use of more than one type of probes or mixtures thereof may also be helpful.

For the purposes of the current work, where we are interested in the relative druggabilty of different mutants/orientations of membrane-bound K-Ras, an inherently less cluttered differential probe occupancy (or density) is most relevant. Both grid-based and distance-based occupancy measures can be used for such an analysis, but we found the latter to be more convenient. The following sections will therefore focus on changes in distance-based probe occupancies.

### Ligand binding sites are modulated by membrane binding

As noted earlier, the current work was motivated in part by the observation that bilayer interactions of H-Ras G12V [54] and K-Ras G12D (Prakash and Gorfe, to be published) involve at least two distinct modes. The prime difference between the modes is the orientation of the catalytic domain with respect to the membrane plane so that, in the case of K-Ras G12D, either helix 3/4 or helix 2 directly contact the bilayer (Fig 1). We wanted to see if these two membrane-binding modes differ in ligand binding potential when assessed by pMD-membrane. To this end, we calculated the difference in atomic probe occupancy between the simulations started from the conformation in Fig 1B (mode 2) and from the conformation in Fig 1A (mode 1): ΔR^conf^ = R^2^ –R^1^. Thus, negative ΔR^conf^ at a given atom means that the atom is more accessible to probes in membrane binding mode 1 than 2 (positive ΔR^conf^ is the opposite).

**Fig 6:**
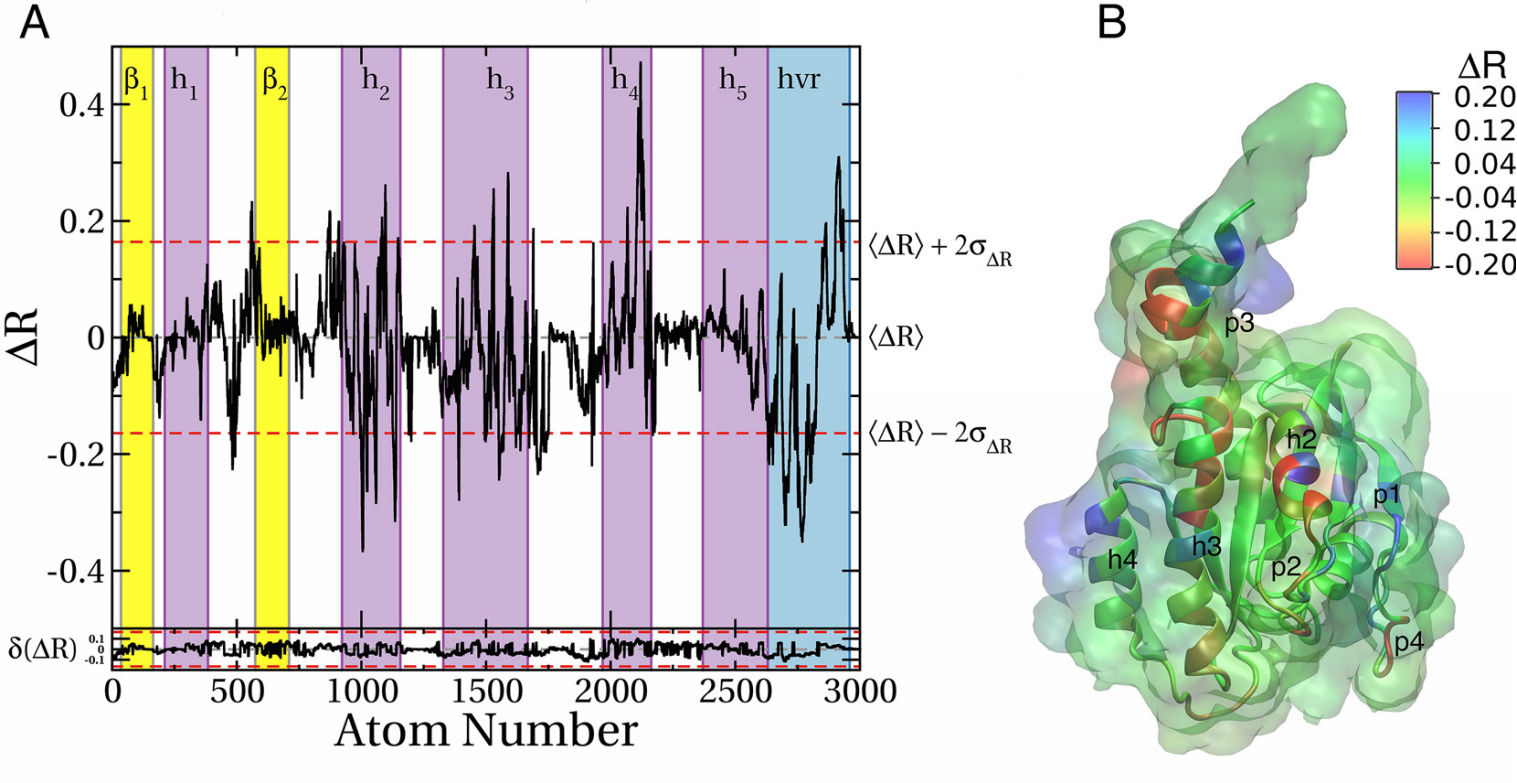
Differences in probe binding between membrane binding modes 1 and 2 of K-Ras G12D. ***(A)*** Profile of the difference in atomic probe occupancy (ΔR) between membrane binding modes 2 and 1. Negative ΔR at a given atom indicates lower probe density in mode 2 than mode 1 while a positive value indicates the opposite. ΔR values outside the upper and lower dashed lines (±2σ) highlight hotspots where significant change in probe binding occurred. Estimated sampling errors of ΔR^conf^ for each atom was calculated using Eq 6 and plotted in the bottom. ***(B)*** Surface of the protein colored by ΔR, where blue indicates sites whose probe binding potential is higher in mode 2 than mode 1; red indicates the opposite.

The data in Fig 6A shows that the two membrane binding modes substantially differ in probe occupancy, particularly at helices h2, h3, h4, the hvr and to a lesser extent between h1 and β2. Coloring the 3D structure by ΔR further shows that the differences are confined to four surface patches (Fig 6B). Three of these patches correspond to previously described pockets, including p1, p3 and part of p2. Pocket p3 is more accessible in mode 1 than in mode 2 (negative ΔR^conf^) while p1 is more accessible in mode 2 than in mode 1. There are some changes in the accessibility of p4 as well. Apart from these pockets, variations in probe accessibility include surface sites that may not be druggable, such as the sharp positive ΔR^conf^ peak on helix 4 arising from the fact that it is engaged with the bilayer in mode 1 but not in mode 2.

Overall, this analysis demonstrates that sites’ accessibility to probe molecules is a function of membrane orientation of the protein, and that pMD-membrane is capable of capturing those differences. We propose that, at least for Ras proteins, ligand accessibility will likewise depend on the details of membrane binding.

### Impact of mutation on druggability of sites

**Fig 7:**
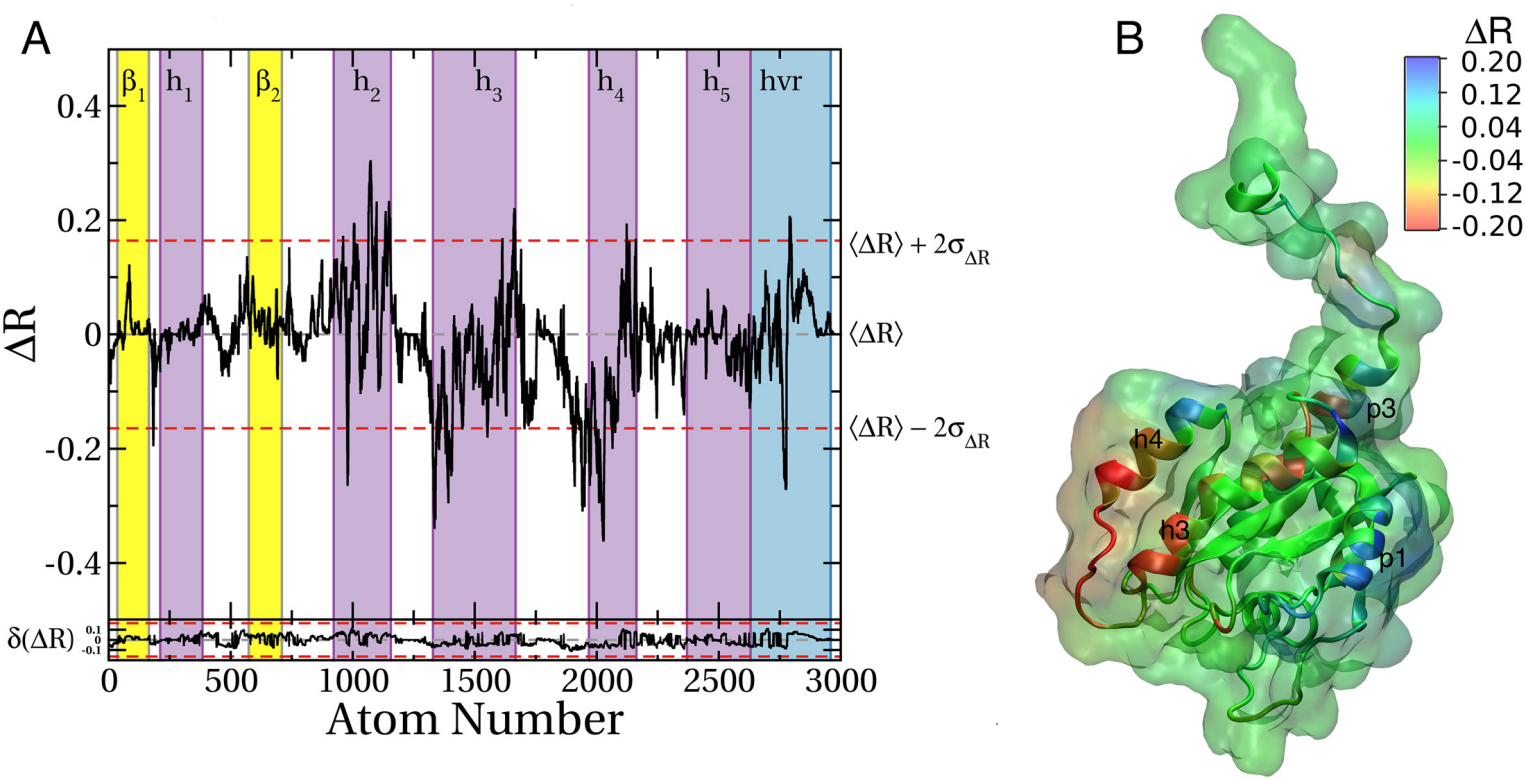
Differences in probe binding between G12D and G13D K-Ras. (A) Same as Fig 6A except that ΔR is the difference in atomic probe occupancies of G12D and G13D K-Ras. Estimated sampling errors of ΔR^seq^ for each atom was calculated using Eq 6 and plotted in the bottom. (B) Same as Fig 6B but using the ΔR values shown in Fig 7A.

In an unpublished study, we observed that the active sites of G12D and G13D K-Ras significantly differ. While the active site of G12D K-Ras is similar to wild type, switch 1 is open and some functionally critical residues, such as Tyr32, have re-oriented in G13D K-Ras. At the functional level, G12D and G13D K-Ras differ in intrinsic GTPase activity [74] and oncogenicity [25]. Therefore, we ran a pMD-membrane simulation on G13D starting from the conformation shown in Fig 1A (mode 1) and calculated ΔR^seq^ = R^G13D^ –R^G12D^. We found major differences in probe accessibility of the two mutants (Fig 7A), indicating that the two simulations started from the same initial configuration have drifted apart, leading to different probe binding propensities. The differences are largely confined to helices 2, 3, and 4 (Fig 7A), representing two surfaces on the 3D structure (Fig 7B). Part of the surface of helix 2 where p1 is located is more accessible in G13D than G12D whereas the region between helices 3 and 4 is significantly more probe-accessible in G12D than G13D. This is despite the fact that these regions are far away from the site of the mutation. We propose that these observations highlight potential differences in the druggability of the two mutants and thereby the possibility of isoform-specific drug leads. We find the region between helices 3 and 4 especially interesting as it might represent a potentially unique new ligand-binding site.

### Analysis of probe orientation

**Fig 8:**
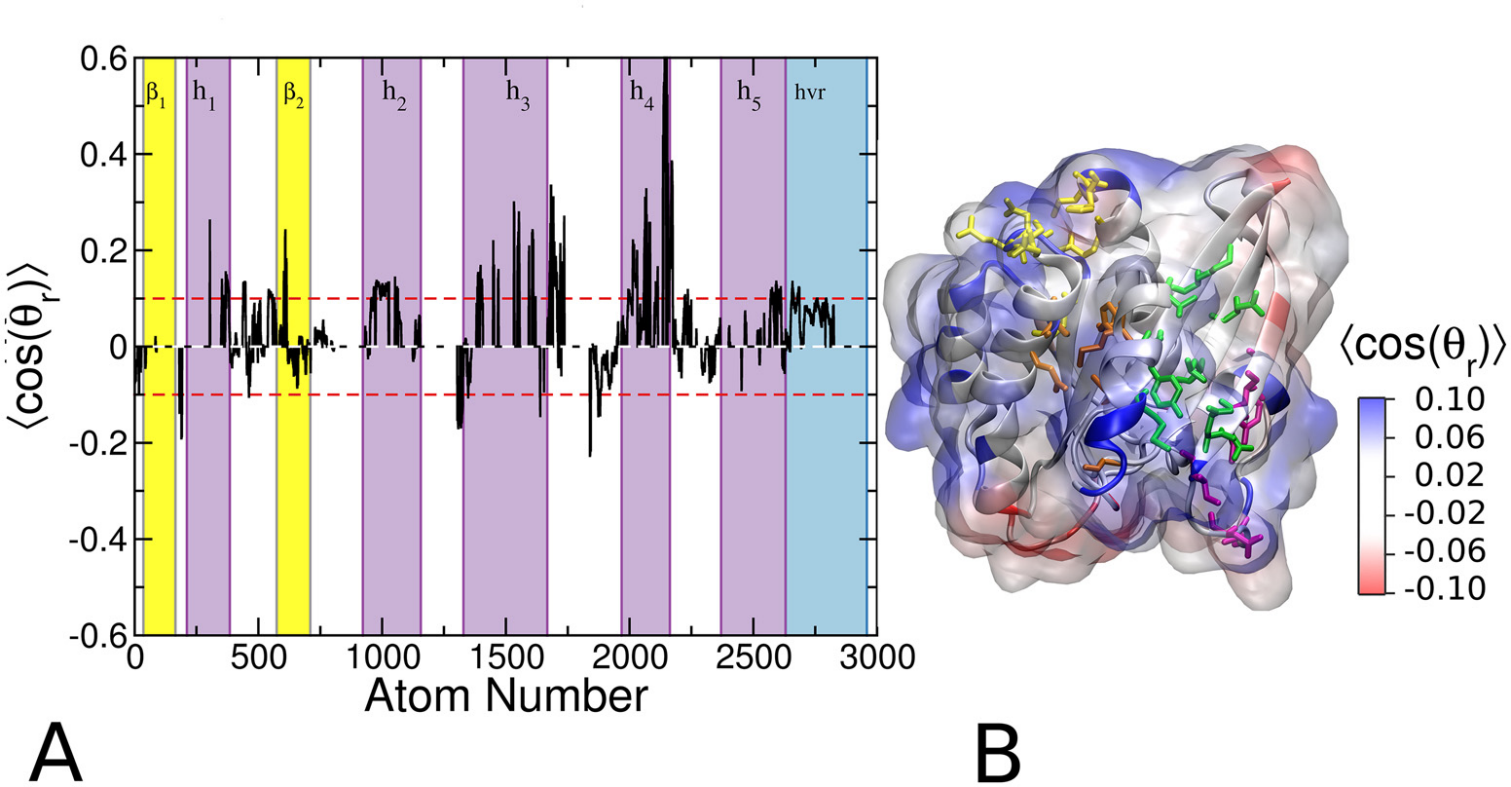
Probe orientation on the surface of G12D K-Ras. ***(A)*** Average values of the probe orientation order parameter <cos(θ_r_)> for G12D mode 1. Note that <cos(θ_r_)> characterizes the orientation of the probes with respect to the surface of the protein at each atom. ***(B)*** Projection of <cos(θ_r_)> onto the 3D structure of the protein, where blue indicates that the probe oxygen is pointing away from the surface of the protein and red the opposite. Residues that define pockets p1, p2, p3 and p4 are shown in green, orange, yellow and purple licorice, respectively.

We have seen that isopropanol has preferred orientations at the glycerol and head group regions of the POPC/POPS bilayer (Figs 2 and 3). This was because the OH functional group of the probe prefers to interact with lipid oxygen atoms which the CH3 groups tend to avoid. For the same reason, interaction of the probes with protein atoms is likely to be polarized so that the OH group points away from hydrophobic surface cavities but points toward polar cavities. Therefore, we wondered if the local orientation of the probe contains information about the polarity/hydrophobicy of individual sites. To check this, we calculated the average orientation of the probe taking into account every protein atom that is in contact with a probe molecule. In this analysis, positive <cos(θ_r_)> indicates that the hydroxyl oxygen points away from the protein (see Methods). We found that the ensemble averaged cos(θ_r_) is positive for the vast majority of the highly probe-accessible surface protein atoms (Fig 8A and 8B), suggesting that the probe-binding sites are mostly hydrophobic and therefore potentially druggable. Negative <cos(θ_r_)> was found only at a couple of surface sites that are unlikely to bind to ligands.

### Conclusion

We introduced a technique referred to as pMD-membrane as a novel approach for the analysis of ligand binding potential of surface cavities in membrane proteins. This represents a major expansion of the scope of probe-based molecular dynamics simulation approaches. The goal of pMD-membrane, and probe-based simulations in general, is to map fragment positions in pockets that may then be used by medicinal chemists to design specific binders. Extensive analysis involving multiple types of co-solvents and multiple drug targets found a strong correlation between probe occupancies and binding affinities of true binders in most cases (refs 7–9). Similarly, we have shown that, following modification of selected vdW interaction terms in the force field, pMD-membrane was able to identify allosteric ligand binding sites (including known binding sites) on the surface-bound K-Ras without any significant effect on the structure and dynamics of the bilayer or the protein. We have also demonstrated that pMD-membrane can capture the impact of conformational changes induced by membrane binding or mutation on the probe accessibility of putative druggable sites. This is important because the ultimate goal of any site identification scheme is to differentiate cryptic binding sites based on changes in size, location or chemical feature. Such changes can result from small differences in protein motion in water versus membrane environments, as well as from mutations, substrate binding, posttranslational modification etc. Our method thus extends the scope of probe-based molecular dynamics simulation in two majors ways: as a novel means by which to find (allosteric) ligand binding sites in membrane proteins and as a tool with which to probe differential ligand accessibility in closely related targets. The method can be easily expanded to any type of probe or mixture of probes through similar modifications of non-bonded terms. Therefore, pMD-membrane and the analysis tools described in this study are applicable to a wide variety of membrane proteins, whether trans-membrane or surface-bound.

## Supporting Information

S1 Fig Effect of modifications of the Lennard-Jones potential. Shown are results from 10 ns MD runs of K-Ras in bilayer with vdW distance of 4 Å (left), 7 Å (middle) and 10 Å (right) between selected atoms of the probe and lipid molecules. For clarity, we show only protein in gray cartoon, bilayer phosphate groups in green and isopropanol in blue sticks. Notice the penetration of isopropanol into the bilayer core in the left panel, their ability to access the protein from every side in the middle panel and their limited access to the side of the protein facing the bilayer in right panel.
(PDF)

S2 Fig Switching function between 6 Å and 8 Å used for the probe occupancy calculation based on Eq 1 in the main text. (PDF)

S3 Fig Convergence and sampling error. (Left) Time evolution of average probe occupancies calculated using Eq 5 for simulations G12D mode 2 (black), G12D mode 1 (red) and G13D (blue). Bold lines indicate 5 ns-running average. (Right) Examples of the dependence of block standard errors (BSE) in R_i_ on block size. Shown here are just a few example curves for selected atoms illustrating convergence (see Methods in the main text for details).
(PDF)

S4 Fig Front and back view of an isosurface (red) that corresponds to a 3.2 M probe concentration. In the left panel, purple represents p3 and grey highlights a previously uncharacterized putative binding site. In the right panel, cyan and green represent pockets p1 and p4, respectively.
(PDF)

## References

[1] Perot S, Sperandio O, Miteva MA, Camproux AC, Villoutreix BO. Druggable pockets and binding site centric chemical space: a paradigm shift in drug discovery. Drug Discov Today 2010;15: 656–667. 10.1016/j.drudis.2010.05.015 20685398

[2] Lu S, Huang W, Zhang J. Recent computational advances in the identification of allosteric sites in proteins. Drug Discov Today 2014;19:1595–1600. 10.1016/j.drudis.2014.07.012 25107670

[3] Allen KN, Bellamacina CR, Ding X, Jeffery CJ, Mattos C, Petsko GA, Ringe D. An Experimental Approach to Mapping the Binding Surfaces of Crystalline Proteins. J Phys Chem 1996;100: 2605–2611.

[4] Dennis S, Kortvelyesi T, Vajda S. Computational mapping identifies the binding sites of organic solvents on proteins. Proc Natl Acad Sci U S A 2002;99: 4290–4295. 11904374 10.1073/pnas.062398499 PMC123641

[5] Hajduk PJ, Huth JR, Fesik SW. Druggability indices for protein targets derived from NMR-based screening data. J Med Chem 2005;48: 2518–2525. 15801841 10.1021/jm049131r

[6] Durrant JD, McCammon JA. Molecular dynamics simulations and drug discovery. BMC Biol 2011;9: 71 10.1186/1741-7007-9-71 22035460 PMC3203851

[7] Seco J, Luque FJ, Barril X. Binding site detection and druggability index from first principles. J Med Chem 2009;52: 2363–2371. 10.1021/jm801385d 19296650

[8] Bakan A, Nevins N, Lakdawala AS, Bahar I. Druggability Assessment of Allosteric Proteins by Dynamics Simulations in the Presence of Probe Molecules. J Chem Theory Comput 2012;8: 2435–2447. 22798729 10.1021/ct300117j PMC3392909

[9] Guvench O, MacKerell AD Jr. Computational Fragment-Based Binding Site Identification by Ligand Competitive Saturation. PLoS Comput Biol 2009;5: e1000435 10.1371/journal.pcbi.1000435 19593374 PMC2700966

[10] Huang D, Caflisch A. Small molecule binding to proteins: affinity and binding/unbinding dynamics from atomistic simulations. ChemMedChem 2011;6: 1578–1580. 10.1002/cmdc.201100237 21674810

[11] Prakash P, Hancock JF, Gorfe AA. Binding hotspots on K-ras: Consensus ligand binding sites and other reactive regions from probe-based molecular dynamics analysis. Proteins 2015;83: 898–909. 10.1002/prot.24786 25740554 PMC4400267

[12] Lexa KW, Carlson HA. Full protein flexibility is essential for proper hot-spot mapping. J Am Chem Soc 2011;133: 200–202. 10.1021/ja1079332 21158470 PMC3081398

[13] Raman EP, Yu W, Guvench O, MacKerell AD. Reproducing Crystal Binding Modes of Ligand Functional Groups Using Site-Identification by Ligand Competitive Saturation (SILCS) Simulations. J Chem Inf Model 2011;51: 877–896. 10.1021/ci100462t 21456594 PMC3090225

[14] Gorfe AA. Mechanisms of allostery and membrane attachment in Ras GTPases: implications for anti-cancer drug discovery. Curr Med Chem 2010;17: 1–9. 19941482 10.2174/092986710789957832

[15] Lundstrom K. Latest development in drug discovery on G protein-coupled receptors. Curr Protein Pept Sci 2006;7: 465–470. 17073697 10.2174/138920306778559403

[16] Prakash P, Gorfe AA. Overview of simulation studies on the enzymatic activity and conformational dynamics of the GTPase Ras. Mol Simul 2014;40: 839–847. 26491216 10.1080/08927022.2014.895000 PMC4610817

[17] Prakash P, Gorfe AA. Lessons from computer simulations of Ras proteins in solution and in membrane. Biochim Biophys Acta 2013;1830: 5211–5218. 10.1016/j.bbagen.2013.07.024 23906604 PMC3825463

[18] Tautermann CS, Seeliger D, Kriegl JM. What can we learn from molecular dynamics simulations for GPCR drug design? Comput Struct Biotechnol J 2015;13: 111–121. 10.1016/j.csbj.2014.12.002 25709761 PMC4334948

[19] Lappano R, Maggiolini M. G protein-coupled receptors: novel targets for drug discovery in cancer. Nat Rev Drug Discov 2011;10: 47–60. 10.1038/nrd3320 21193867

[20] Prakash P, Gorfe AA. Phosphatidylcholine attenuates aggregation of nonsteroidal anti-inflammatory drugs with bile acid. Biochemistry 2013;52: 7461–7469. 10.1021/bi400723r 24066846

[21] Prakash P, Sayyed-Ahmad A, Zhou Y, Volk DE, Gorenstein DG, Dial E, Lichtenberger LM, Gorfe AA. Aggregation behavior of ibuprofen, cholic acid and dodecylphosphocholine micelles. Biochim Biophys Acta 2012; 1818: 3040–3047. 10.1016/j.bbamem.2012.07.029 22885171 PMC3478136

[22] Sayyed-Ahmad A, Lichtenberger LM, Gorfe AA. Structure and Dynamics of Cholic Acid and Dodecylphosphocholine—Cholic Acid Aggregates. Langmuir 2010;26: 13407–13414. 10.1021/la102106t 20695585 PMC2924285

[23] Boggara MB, Krishnamoorti R. Partitioning of nonsteroidal antiinflammatory drugs in lipid membranes: a molecular dynamics simulation study. Biophys J 2010;98: 586–595. 10.1016/j.bpj.2009.10.046 20159155 PMC2820636

[24] Soares ARM, Thanaiah Y, Taniguchi M, Lindsey JS. Aqueous-membrane partitioning of [small beta]-substituted porphyrins encompassing diverse polarity. New J Chem 2013;37: 1087–1097.

[25] Cox AD, Der CJ. Ras history: The saga continues. Small GTPases 2010;1: 2–27. 21686117 10.4161/sgtp.1.1.12178 PMC3109476

[26] Barbacid M. Ras Genes. Annu Rev Biochem 1987;56: 779–827. 3304147 10.1146/annurev.bi.56.070187.004023

[27] Karnoub AE, Weinberg RA. Ras oncogenes: split personalities. Nat Rev Mol Cell Biol 2008; 9: 517–531. 10.1038/nrm2438 18568040 PMC3915522

[28] Bos JL. Ras oncogenes in human cancer: a review. Cancer Res 1989; 49: 4682–4689. 2547513

[29] Prior IA, Lewis PD, Mattos C. A comprehensive survey of Ras mutations in cancer. Cancer Res 2012;72: 2457–2467. 10.1158/0008-5472.CAN-11-2612 22589270 PMC3354961

[30] Cox AD, Fesik SW, Kimmelman AC, Luo J, Der CJ. Drugging the undruggable RAS: Mission Possible? Nat Rev Drug Discov 2014;13: 828–851. 10.1038/nrd4389 25323927 PMC4355017

[31] Wang W, Fang G, Rudolph J. Ras inhibition via direct Ras binding—is there a path forward? Bioorg Med Chem Lett 2012;22: 5766–5776. 10.1016/j.bmcl.2012.07.082 22902659

[32] Maurer T, Garrenton LS, Oh A, Pitts K, Anderson DJ, Skelton NJ, Fauber BP, Pan B, Malek S, Stokoe D, Ludlam MJ, Bowman KK, Wu J, Giannetti AM, Starovasnik MA, Mellman I, Jackson PK, Rudolph J, Wang W, Fang G. Small-molecule ligands bind to a distinct pocket in Ras and inhibit SOS-mediated nucleotide exchange activity. Proc Natl Acad Sci U S A 2012;109: 5299–5304. 10.1073/pnas.1116510109 22431598 PMC3325706

[33] Sun Q, Burke JP, Phan J, Burns MC, Olejniczak ET, Waterson AG, Lee T, Rossanese OW, Fesik SW. Discovery of small molecules that bind to K-Ras and inhibit Sos-mediated activation. Angew Chem Int Ed Engl 2012;51:6140–6143. 10.1002/anie.201201358 22566140 PMC3620661

[34] Shima F, Yoshikawa Y, Ye M, Araki M, Matsumoto S, Liao J, Hu L, Sugimoto T, Ijiri Y, Takeda A, Nishiyama Y, Sato C, Muraoka S, Tamura A, Osoda T, Tsuda K.-I, Miyakawa T, Fukunishi H, Shimada J, Kumasaka T, Yamamoto M, Kataoka T. In silico discovery of small-molecule Ras inhibitors that display antitumor activity by blocking the Ras–effector interaction. Proc Natl Acad Sci U S A 2013;110:8182–8187. 10.1073/pnas.1217730110 23630290 PMC3657810

[35] Rosnizeck IC, Graf T, Spoerner M, Tränkle J, Filchtinski D, Herrmann C, Gremer L, Vetter IR, Wittinghofer A, König B, Kalbitzer HR. Stabilizing a Weak Binding State for Effectors in the Human Ras Protein by Cyclen Complexes. Angew Chem Int Ed Engl 2010;49:3830–3833. 10.1002/anie.200907002 20401883

[36] Rosnizeck IC, Spoerner M, Harsch T, Kreitner S, Filchtinski D, Herrmann C, Engel D, König B, Kalbitzer HR. Metal–Bis(2-picolyl)amine Complexes as State 1(T) Inhibitors of Activated Ras Protein. Angew Chem Int Ed Engl 2012;51:10647–10651. 10.1002/anie.201204148 22996816

[37] Ostrem JM, Peters U, Sos ML, Wells JA, Shokat KM. K-Ras(G12C) inhibitors allosterically control GTP affinity and effector interactions. Nature 2013;503:548–551. 10.1038/nature12796 24256730 PMC4274051

[38] Hocker HJ, Cho KJ, Chen CY, Rambahal N, Sagineedu SR, Sharri K, Stanslas J, Hancock JF, Gorfe AA. Andrographolide derivatives inhibit guanine nucleotide exchange and abrogate oncogenic Ras function. Proc Natl Acad Sci U S A 2013; 110:10201–10206. 10.1073/pnas.1300016110 23737504 PMC3690838

[39] Omerovic J, Prior IA. Compartmentalized signalling: Ras proteins and signalling nanoclusters. FEBS J 2009;276: 1817–1825. 10.1111/j.1742-4658.2009.06928.x 19243428

[40] Prior IA, Hancock JF. Compartmentalization of Ras proteins. J Cell Sci 2001;114: 1603–1608. 11309191 10.1242/jcs.114.9.1603

[41] Hancock JF, Prior IA. Electron microscopic imaging of Ras signaling domains. Methods 2005;37: 165–172. 16288888 10.1016/j.ymeth.2005.05.018 PMC3351669

[42] Aran V, Prior IA. Compartmentalized Ras signaling differentially contributes to phenotypic outputs. Cell Signal 2013;25: 1748–1753. 10.1016/j.cellsig.2013.05.004 23707528 PMC3776226

[43] Gorfe AA, Grant BJ, McCammon JA. Mapping the nucleotide and isoform-dependent structural and dynamical features of Ras proteins. Structure 2008;16: 885–896. 10.1016/j.str.2008.03.009 18547521 PMC2519881

[44] Almoguera C, Shibata D, Forrester K, Martin J, Arnheim N, et al Most human carcinomas of the exocrine pancreas contain mutant c-K-ras genes. Cell 1988;53: 549–554. 2453289 10.1016/0092-8674(88)90571-5

[45] Jones S, Zhang X, Parsons DW, Lin JC, Leary RJ, et al Core signaling pathways in human pancreatic cancers revealed by global genomic analyses. Science 2008;321: 1801–1806. 10.1126/science.1164368 18772397 PMC2848990

[46] Forrester K, Almoguera C, Han K, Grizzle WE, Perucho M. Detection of high incidence of K-ras oncogenes during human colon tumorigenesis. Nature 1987;327: 298–303. 2438556 10.1038/327298a0

[47] Riely GJ, Marks J, Pao W. KRAS mutations in non-small cell lung cancer. Proc Am Thorac Soc 2009;6: 201–205. 10.1513/pats.200809-107LC 19349489

[48] van 't Veer LJ, Burgering BM, Versteeg R, Boot AJ, Ruiter DJ, et al N-ras mutations in human cutaneous melanoma from sun-exposed body sites. Mol Cell Biol 1989;9: 3114–3116. 2674680 10.1128/mcb.9.7.3114-3116.1989 PMC362784

[49] Ball NJ, Yohn JJ, Morelli JG, Norris DA, Golitz LE, et al Ras mutations in human melanoma: a marker of malignant progression. J Invest Dermatol 1994;102: 285–290. 8120410 10.1111/1523-1747.ep12371783

[50] Luo D, Liu QF, Gove C, Naomov N, Su JJ, et al Analysis of N-ras gene mutation and p53 gene expression in human hepatocellular carcinomas. World J Gastroenterol 1998; 4: 97–99. 11819246 10.3748/wjg.v4.i2.97 PMC4688651

[51] Burchill SA, Neal DE, Lunec J. Frequency of H-ras mutations in human bladder cancer detected by direct sequencing. Br J Urol 1994;73: 516–521. 8012773 10.1111/j.1464-410x.1994.tb07636.x

[52] Castro P, Soares P, Gusmao L, Seruca R, Sobrinho-Simoes M. H-RAS 81 polymorphism is significantly associated with aneuploidy in follicular tumors of the thyroid. Oncogene 2006;25: 4620–4627. 16532025 10.1038/sj.onc.1209491

[53] Marcus K, Mattos C. Direct Attack on RAS: Intramolecular Communication and Mutation-Specific Effects. Clin Cancer Res 2015;21: 1810–1818. 10.1158/1078-0432.CCR-14-2148 25878362

[54] Gorfe AA, Hanzal-Bayer M, Abankwa D, Hancock JF, McCammon JA. Structure and dynamics of the full-length lipid-modified H-Ras protein in a 1,2-dimyristoylglycero-3-phosphocholine bilayer. J Med Chem 2007;50: 674–684. 17263520 10.1021/jm061053f

[55] Abankwa D, Hanzal-Bayer M, Ariotti N, Plowman SJ, Gorfe AA, et al A novel switch region regulates H-ras membrane orientation and signal output. EMBO J 2008;27: 727–735. 10.1038/emboj.2008.10 18273062 PMC2265749

[56] Abankwa D, Gorfe AA, Inder K, Hancock JF. Ras membrane orientation and nanodomain localization generate isoform diversity. Proc Natl Acad Sci U S A 2010;107: 1130–1135. 10.1073/pnas.0903907107 20080631 PMC2824305

[57] Abankwa D, Gorfe AA, Hancock JF. Ras nanoclusters: molecular structure and assembly. Semin Cell Dev Biol 2007;18: 599–607. 17897845 10.1016/j.semcdb.2007.08.003 PMC2761225

[58] Janosi L, Gorfe AA. Segregation of negatively charged phospholipids by the polycationic and farnesylated membrane anchor of Kras. Biophys J 2010;99: 3666–3674 10.1016/j.bpj.2010.10.031 21112291 PMC2998625

[59] Darden T, York D, Pedersen L. Particle mesh Ewald: An N⋅log(N) method for Ewald sums in large systems. J Chem Phys 1993;98: 10089–10092.

[60] Ryckaert JP, Ciccotti G, Berendsen HJC. Numerical integration of the Cartesian Equations of Motion of a System with Constraints: Molecular Dynamics of n-Alkanes. J Comput Phys 1977;23: 327–341.

[61] Phillips JC, Braun R, Wang W, Gumbart J, Tajkhorshid E, et al Scalable molecular dynamics with NAMD. J Comput Chem 2005; 26: 1781–1802. 16222654 10.1002/jcc.20289 PMC2486339

[62] MacKerell AD, Bashford D, Bellott M, Dunbrack RL, Evanseck JD, Field MJ, Fischer S, Gao J, Guo H, Ha S, Joseph-McCarthy D, Kuchnir L, Kuczera K, Lau FT, Mattos C, Michnick S, Ngo T, Nguyen DT, Prodhom B, Reiher WE, Roux B, Schlenkrich M, Smith JC, Stote R, Straub J, Watanabe M, Wiórkiewicz-Kuczera J, Yin D, Karplus M. All-Atom Empirical Potential for Molecular Modeling and Dynamics Studies of Proteins. J Phys Chem B 1998;102:586–3616. 10.1021/jp973084f 24889800

[63] Klauda JB, Venable RM, Freites JA, O'Connor JW, Tobias DJ, et al Update of the CHARMM all-atom additive force field for lipids: validation on six lipid types. J Phys Chem B 2010;114: 7830–7843. 10.1021/jp101759q 20496934 PMC2922408

[64] Humphrey W, Dalke A, Schulten K. VMD: visual molecular dynamics. J Mol Graph 1996;14: 33–38. 8744570 10.1016/0263-7855(96)00018-5

[65] Grossfield A, Zuckerman DM. Quantifying uncertainty and sampling quality in biomolecular simulations. Annu Rep Comput Chem 2009;5: 23–48. 20454547 10.1016/S1574-1400(09)00502-7 PMC2865156

[66] Stephen AG, Esposito D, Bagni RK, McCormick F. Dragging ras back in the ring. Cancer Cell 2014;25: 272–281. 10.1016/j.ccr.2014.02.017 24651010

[67] Fenimore PW, Frauenfelder H, McMahon BH, Parak FG. Slaving: solvent fluctuations dominate protein dynamics and functions. Proc Natl Acad Sci U S A 2002;99: 16047–16051. 12444262 10.1073/pnas.212637899 PMC138562

[68] Prakash P, Sayyed-Ahmad A, Gorfe AA. The role of conserved waters in conformational transitions of Q61H K-ras. PLoS Comput Biol 2012;8: e1002394 10.1371/journal.pcbi.1002394 22359497 PMC3280954

[69] Buhrman G, O′Connor C, Zerbe B, Kearney BM, Napoleon R, et al Analysis of Binding Site Hot Spots on the Surface of Ras GTPase. J Mol Biol 2011;413: 773–789. 10.1016/j.jmb.2011.09.011 21945529 PMC3247908

[70] Grant BJ, Lukman S, Hocker HJ, Sayyah J, Brown JH, et al Novel allosteric sites on Ras for lead generation. PLoS One 2011;6: e25711 10.1371/journal.pone.0025711 22046245 PMC3201956

[71] Halgren TA. Identifying and characterizing binding sites and assessing druggability. J Chem Inf Model 2009;49: 377–389. 10.1021/ci800324m 19434839

[72] Schmidtke P, Bidon-Chanal A, Luque FJ, Barril X. MDpocket: open-source cavity detection and characterization on molecular dynamics trajectories. Bioinformatics 2011;27: 3276–3285. 10.1093/bioinformatics/btr550 21967761

[73] Durrant JD, Lindert S, McCammon JA. AutoGrow 3.0: an improved algorithm for chemically tractable, semi-automated protein inhibitor design. J Mol Graph Model 2013;44: 104–112. 10.1016/j.jmgm.2013.05.006 23792207 PMC3842281

[74] Smith MJ, Neel BG, Ikura M. NMR-based functional profiling of RASopathies and oncogenic RAS mutations. Proc Natl Acad Sci U S A 2013;110: 4574–4579. 10.1073/pnas.1218173110 23487764 PMC3607025

