# Supplementary material for: pMD-Membrane: A Method for Ligand Binding Site Identification in Membrane-Bound Proteins
Source: PLoS Comput Biol. 2015 Oct 27;11(10):e1004469. doi: 10.1371/journal.pcbi.1004469 (PMC4623977; doi:10.1371/journal.pcbi.1004469)
Supplement: S1 Fig — Shown are results from 10 ns MD runs of K-Ras in bilayer with vdW distance of 4 Å (left), 7 Å (middle) and 10 Å (right) between selected atoms of the probe and lipid molecules. For clarity, we show only protein in gray cartoon, bilayer phosphate groups in green and isopropanol in blue sticks. Notice the penetration of isopropanol into the bilayer core in the left panel, their ability to access the protein from every side in the middle panel and their limited access to the side of the protein facing the bilayer in right panel. (PDF) [file pcbi.1004469.s001.pdf]

Supplementary Information

pMD-membrane: A method for ligand binding site identification in membrane-bound proteins

Priyanka Prakash, Abdallah Sayyed-Ahmad and Alemayehu A. Gorfe*

University of Texas Health Science Center at Houston, Department of Integrative Biology and Pharmacology, 6431 Fannin St., Houston, Texas 77030

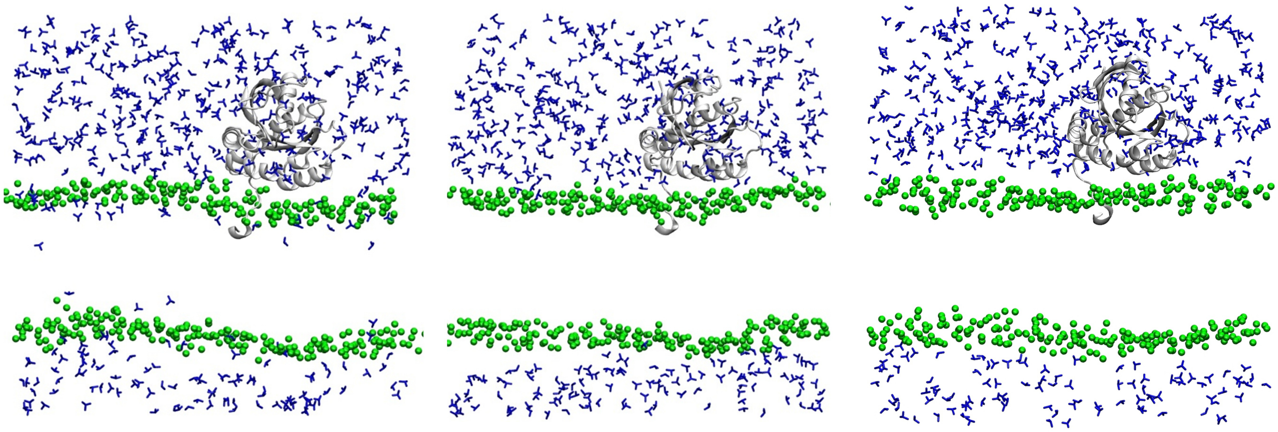

Effect modifications of Lennard-Jones potentials on probe binding. Shown are results from 10ns MD runs of K-Ras in bilayer with vdW distance of 4 Å (left), 7 Å (middle) and 10 Å (right) between selected atoms of the probe and lipid molecules. For clarity, we show only protein in gray cartoon, bilayer phosphate groups in green and isopropanol in blue sticks. Notice the penetration of isopropanol into the bilayer core in the left panel, their ability to access the protein from every side in the middle panel and their limited access to the side of the protein facing the bilayer in right panel.
