# Supplementary material for: pMD-Membrane: A Method for Ligand Binding Site Identification in Membrane-Bound Proteins
Source: PLoS Comput Biol. 2015 Oct 27;11(10):e1004469. doi: 10.1371/journal.pcbi.1004469 (PMC4623977; doi:10.1371/journal.pcbi.1004469)
Supplement: S2 Fig — (PDF) [file pcbi.1004469.s002.pdf]

Supplementary Information

pMD-membrane: A method for ligand binding site identification in membrane-bound proteins

Priyanka Prakash, Abdallah Sayyed-Ahmad and Alemayehu A. Gorfe*

University of Texas Health Science Center at Houston, Department of Integrative Biology and Pharmacology, 6431 Fannin St., Houston, Texas 77030

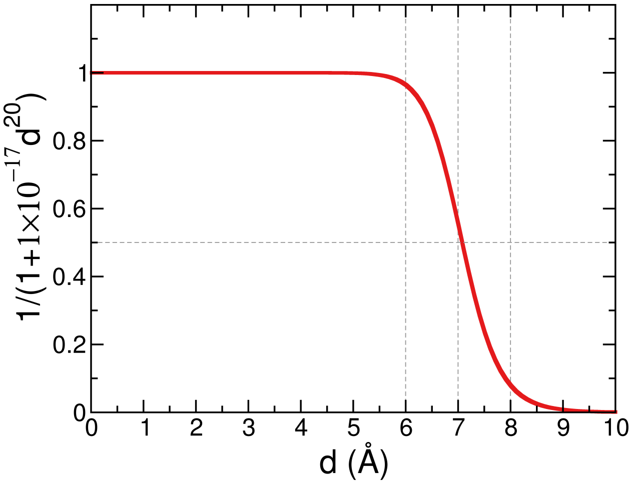

Figure S2: Switching function between 6 Å and 8 Å used for the probe occupancy calculation based on equation 1 in the main text.
