# Supplementary material for: pMD-Membrane: A Method for Ligand Binding Site Identification in Membrane-Bound Proteins
Source: PLoS Comput Biol. 2015 Oct 27;11(10):e1004469. doi: 10.1371/journal.pcbi.1004469 (PMC4623977; doi:10.1371/journal.pcbi.1004469)
Supplement: S3 Fig — (Left) Time evolution of average probe occupancies calculated using Eq 5 for simulations G12D mode 2 (black), G12D mode 1 (red) and G13D (blue). Bold lines indicate 5 ns-running average. (Right) Examples of the dependence of block standard errors (BSE) in Ri on block size. Shown here are just a few example curves for selected atoms illustrating convergence (see Methods in the main text for details). (PDF) [file pcbi.1004469.s003.pdf]

Supplementary Information

pMD-membrane: A method for ligand binding site identification in membrane-bound proteins

Priyanka Prakash, Abdallah Sayyed-Ahmad and Alemayehu A. Gorfe*

University of Texas Health Science Center at Houston, Department of Integrative Biology and Pharmacology, 6431 Fannin St., Houston, Texas 77030

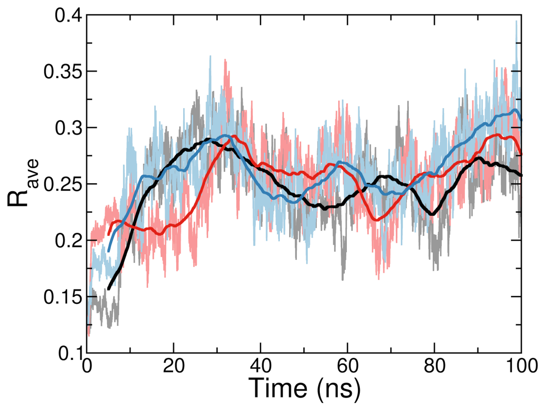

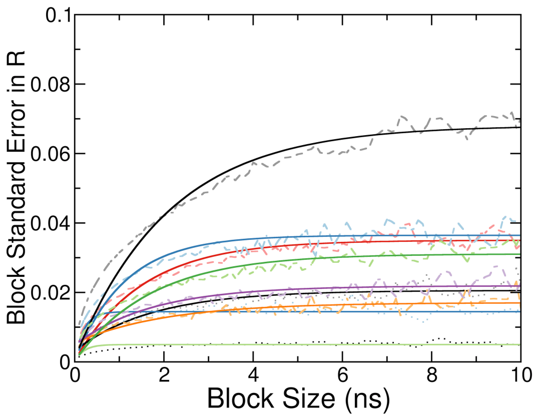

Figure S3: Convergence and sampling error. (Left) Time evolution of average probe occupancies calculated using equation 5 for simulations G12D mode 2 (black), G12D mode 1 (red) and G13D (blue). Bold lines indicate 5 ns-running average. (Right) Examples of the dependence of block standard errors (BSE) in R_i on block size. Shown here are just a few example curves for selected atoms illustrating atoms (see Methods in the main text for details).
