# Supplementary material for: pMD-Membrane: A Method for Ligand Binding Site Identification in Membrane-Bound Proteins
Source: PLoS Comput Biol. 2015 Oct 27;11(10):e1004469. doi: 10.1371/journal.pcbi.1004469 (PMC4623977; doi:10.1371/journal.pcbi.1004469)
Supplement: S4 Fig — In the left panel, purple represents p3 and grey highlights a previously uncharacterized putative binding site. In the right panel, cyan and green represent pockets p1 and p4, respectively. (PDF) [file pcbi.1004469.s004.pdf]

Supplementary Information

pMD-membrane: A method for ligand binding site identification in membrane-bound proteins

Priyanka Prakash, Abdallah Sayyed-Ahmad and Alemayehu A. Gorfe*

University of Texas Health Science Center at Houston, Department of Integrative Biology and Pharmacology, 6431 Fannin St., Houston, Texas 77030

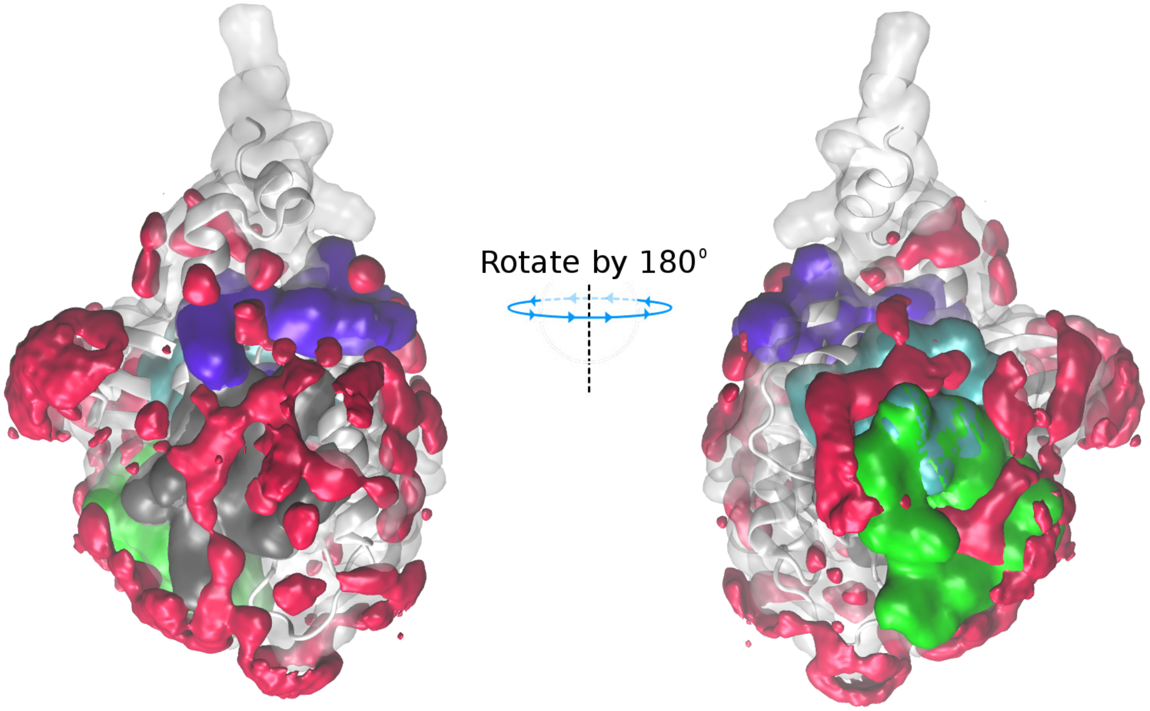

Figure S4: Front and back view of an isosurface (red) that corresponds to a 3.2 M probe concentration. In the left panel, purple represents p3 and grey highlights a previously uncharacterized putative binding site. In the right panel, cyan and green represent pockets p1 and p4, respectively.
